# The Development of Adolescent Social Cognition

**DOI:** 10.1111/j.1749-6632.2009.04509.x

**Published:** 2009-06-24

**Authors:** Stephanie Burnett, Sarah-Jayne Blakemore

**Affiliations:** University College London, Institute of Cognitive NeuroscienceLondon, United Kingdom

**Keywords:** adolescence, social brain, development

## Abstract

Adolescence has long been considered a turbulent time; beginning with large changes in hormonal levels and consequent bodily changes, as well as changes in behavior. Recently, neuroscience studies have contributed to this picture of turbulence. We now know that the brain undergoes profound transformation during the teenage years. This paper focuses on how the social brain—the network of brain regions involved in understanding other people and self-awareness—develops during adolescence.

## Introduction

Adolescence is defined in humans as the period of psychological and social transition between childhood and adulthood. The beginning of adolescence, around the onset of puberty, is characterized by dramatic changes in hormone levels and, as a result, in physical appearance. This period of life is also characterized by the continued development of social abilities and behavior as well as neuroanatomical maturation within regions of the brain involved in social cognition.^1^ Recent advances in cognitive neuroscience are allowing us to begin to probe the links between unfolding adolescent social cognition and its physical basis in the brain.

## Milestones in Social Ability from Birth to Adulthood

Human social preferences are apparent at a very early age. At only a few weeks after birth, infants direct more smiles toward their caregiver and other humans than at inanimate objects, indicating that they differentiate between social and nonsocial beings. From around 1 year, infants deliberately engage and redirect the attention of their caregiver by pointing or vocalizing. By about 2.5 years, children implement complex social tactics, such as teasing, lying, and saving face (bravado).^2^ Over the next few years, individuals learn to use these social tactics flexibly in different social situations. For example, children aged 5 or 6 can use deception to protect other people's feelings (telling “white” lies) in contrast to younger children who mainly use deception for self-serving reasons (e.g., to avoid punishment). A growing understanding of the self-conscious emotions (such as embarrassment, guilt, and pride) at around the same age indicates that children are beginning to explicitly take other people's feelings into account in their emotional reactions to situations.^3^ By middle childhood, concepts of fairness and justice show through in an emerging tendency to share resources equally.^4^

The understanding of how social abilities develop during late childhood and adolescence is much less complete. Although social psychology research on adolescence has been fruitful since the 1970s,^5^ research into adolescent social *cognitive* development, that is, the component mental processes that underlie complex social behaviors, is comparatively younger.^6^–^9^ However, accumulating evidence points to the continuing development of the ability to read emotion in faces and of proficiency in taking on other emotional perspectives (stepping into someone else's shoes).^1^

Another important social ability, the ability to sometimes decide to ignore what others think you should do (resisting peer influence), unfolds during the adolescent years. Steinberg and Monahan conducted a large study in which 3600 male and female children, adolescents, and adults completed a questionnaire asking how likely they would be to do a variety of good, bad, or neutral actions based on whether other people were doing the same. It was found that self-reported resistance to peer influence (RPI) increased steadily between the middle and late teens (ages 14 to 18).^10^ Another study was conducted by Gardner and Steinberg to look at the effects of developing RPI on risk-taking behavior. A laboratory study was conducted in which adolescents (aged 13–16), youths (aged 18–22), and adults (24+) played a car-driving video game either alone or with two friends present.^11^ It was found that in the presence of friends the adolescents (and to a lesser extent the youths) took many more risks while driving, for example, failing to stop at a yellow traffic light. Levels of risk taking did not increase for adult participants if their friends were watching, and when adolescents were playing alone they showed the same level of risk taking as did adults. Recently, it has been shown that this laboratory game has parallels in real life. The Association of British Insurers reported in 2008 that teenagers are three times more likely to have a fatal crash when driving with peers compared to when driving alone.^12^

The onset of adolescence also marks a change in patterns of social behavior. Teenagers begin to enjoy the company of their friends more and to spend more time with them (and consequently less time with their families). During the time spent together, teenagers begin to share their worries, secrets, and ambitions more than they did as children. A more fully integrated social identity emerges, with participation in relationships at different levels—from intimate friendships and romantic attachments, to semiflexible cliques of less than 10 members, to large crowds of individuals who share distinct fashions and interests but are not necessarily all individually acquainted.^13^ At the end of adolescence, an individual is expected to emerge as a socially capable adult.

## Brain Development during Adolescence

Until relatively recently, it was widely held that the brain was anatomically mature early in life. A small number of studies published in the late 1960s and 1970s, using post-mortem brain samples,^14^,^15^ suggested that the brain continues to develop during adolescence. However, it was generally assumed that changes in social behavior during the teens were a result of hormones, social experience, and the changing social environment. These factors are undoubtedly important. However, neuroanatomical development, which occurs throughout the teenaged years, may also play a role.

Results from large magnetic resonance imaging (MRI) studies looking at brain development across the lifespan indicate that brain regions involved in social cognition undergo protracted development throughout adolescence.^16^–^19^ In the frontal and parietal lobes, gray matter increases in volume during childhood, reaching its peak at around puberty onset. This is followed by gray matter thinning during the remainder of adolescence. This is in contrast to basic sensory regions of the brain in which peak gray matter volume is attained during childhood (for reviews, see refs. 1 and 20). It has been suggested that the regional increases in gray matter volume up to and around puberty are a result of synaptic proliferation (synaptogenesis) and that subsequent gray matter thinning reflects the elimination or “pruning” of synapses, as has been observed in post-mortem brain samples.^15^,^21^,^22^ These changes would be expected to result in more finely tuned neural circuits, which will respond optimally to the task in hand. Among the brain regions that undergo these changes in gray matter volume during the adolescent years is the prefrontal cortex, a region involved in higher cognitive abilities, including social cognition and the planned control of behavior. This suggests that the high-level abilities subsumed by these late-maturing regions may continue to develop during the adolescent years.

Another major neuroanatomical change that has been observed using MRI is a linear increase in white matter volume, which occurs across the brain throughout childhood and adolescence (and, indeed, into the 20s). This increase in white matter volume is thought to reflect ongoing maturation of neuronal axons, for example, myelination and/or increasing axonal caliber.^14^,^23^,^20^ These processes might be expected to result in faster and more efficient neuronal signaling.

## Functional Imaging of the Adolescent Social Brain

In the past decade, cognitive neuroscientists have used functional MRI (fMRI) to investigate brain activity during social cognition tasks in adolescent participants. These studies have revealed consistent differences in brain activity between adolescents and adults.

**Figure 1:**
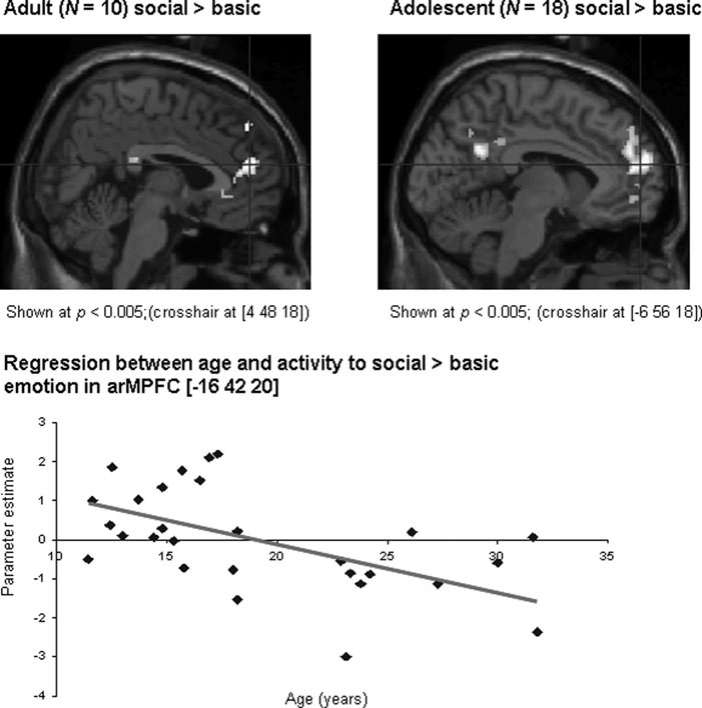
Main effect of social versus basic emotion in adult (*left*) and adolescent (*right*) groups: anterior rostral medial prefrontal cortex (arMPFC) is significantly active in both. Graph (*bottom*) shows the negative correlation between age and activity in arMPFC to social versus basic emotion at the coordinate for which there was a significant group by emotion interaction (see Burnett *et al*., 2009).

In one of these fMRI studies, 18 adolescent volunteers and 10 adults were scanned as they read sentences describing situations in which social or “basic” emotions would be felt.^24^ Social emotions, such as embarrassment or guilt, are emotions that require the consideration of other people's beliefs, feelings, or desires (their “mental states”). For example, embarrassment is felt when you believe that someone judges your actions as foolish, and guilt is experienced when you become aware that someone is suffering because of your actions. In contrast, basic or “gut” emotions, such as pure disgust or pure fear, are all about *you*—and your immediate visceral reactions. Basic emotions do not require you to think about other people's mental states. In this study, components of the so-called “mentalizing system,” comprising anterior rostral medial prefrontal cortex (arMPFC), the posterior superior temporal sulcus at the temporo-parietal junction (pSTS/TPJ), and the anterior temporal lobe (ATL), showed greater activity in social relative to basic emotions in both age groups (see Fig. 1, top).^25^,^26^ However, when activity was compared between age groups, it was found that adolescents activated arMPFC, a brain region involved in mental state representation,^25^ more strongly than did adults for social relative basic emotions (see Fig. 1, bottom). In contrast, adults activated left ATL more strongly than did adolescents in this contrast. ATL is a brain region thought to store social-emotional semantic information.^27^

Another important aspect of social cognition is the ability to understand *yourself*.^28^ This allows you to work out how other people see you and perhaps adjust your behavior depending on the social situation you are in. In a recent fMRI study of self-knowledge, 12 children (aged 10) and 12 adults underwent brain scanning while they judged whether a series of statements, such as “I like to read just for fun,” applied to them.^29^ In another experiment, 19 teenagers and 11 adults were scanned in fMRI as they tried to work out what they would do in different situations (e.g., “If you were bored on a Friday night, would you find out what was on at the cinema?”).^30^ In both experiments, the older children and adolescents activated arMPFC more strongly than the adults, a similar result to that in the social emotion study. Together, these studies collectively suggest that adolescents use brain regions for social cognition differently than do adults, in a variety of situations that require social understanding.

There are a number of plausible explanations for these developmental differences in functional activity within social brain regions. One possible explanation has to do with neuroanatomical development. It could be the case that adolescents activate these developing social brain regions more strongly than adults because the less efficient neural circuits need more oxygen and energy to power them. This might mean that adolescents can do just as well as adults in certain tasks requiring social understanding, but that parts of their brain require more energy to do so. Another possibility is that adolescents are actually using different cognitive strategies to approach social tasks. Perhaps teenagers are still “working out” social situations as a result of accumulating experience or developing social skills. This may mean that they require more effortful, online, social cognitive processing. With age, processing may become less effortful, more automatic, and perhaps more reliant on stored social knowledge. An unexplored implication of this could be that the period of life when arMPFC and other social brain regions are still developing—the teens and early 20s—might be a period of particular open mindedness to new ideas and different types of people.

## Social and Nonsocial Intelligence

At this point, it is worthwhile considering that real-life social behavior relies on many component processes that are not specifically social. These are the cool, forward-thinking processes known as the “executive functions” that allow you to exert control over your behavior and plan ahead. There is evidence that some of these executive functions are still maturing during the teenaged years,^31^ and this may aid the development of adult social behavior. For example, social situations will run more smoothly if you can regulate your immediate emotional reactions (e.g., remaining calm when somebody says something to make you feel angry), focus on the task in hand (e.g., comforting a friend) by resisting temptations (e.g., to ask a nosy question), or keep track of several contingent facts at once (e.g., “If he just said this, when she said that yesterday, what she *really* meant was…”). These abilities, which are useful in both social and nonsocial situations, develop throughout the teens. At the same time, parts of the prefrontal cortex that enable these executive abilities continue to mature.

## Conclusions and Implications

There are many factors responsible for the complex changes in social behavior and self-awareness that take place during the teenaged years. Hormones, genes, and the psychosocial impact of the physical changes of puberty undoubtedly contribute, as do an individual's steadily accumulating experiences with different people and social situations. And although science has little to say on this issue, the day-to-day decisions a teenager chooses to make must surely alter the shape of social awareness and behavior in the adult.

Recently, brain imaging experiments have shown that these changes in social cognition post puberty are also related to brain development. In brain regions such as arMPFC, which is involved in representing mental states, gray and white matter continue to mature throughout the teenaged years. These maturational changes are thought to result in faster and more efficient brain circuits, which will respond more appropriately to the tasks they perform. Another recent discovery is that activity during social cognition tasks differs between adolescence and adulthood. Specifically, adolescents show greater activity within arMPFC than do adults. Whether this means adolescents are approaching social situations using different cognitive strategies, or whether it is a side effect of anatomical brain development in the absence of cognitive change, is not yet known.
